# Physical activity measurement tools among college students in intervention studies: A systematic review

**DOI:** 10.1371/journal.pone.0321593

**Published:** 2025-04-10

**Authors:** Sanying Peng, Ahmad Zamri Khairani, Abubakar Rabiu Uba, Fang Yuan

**Affiliations:** 1 Department of Physical Education, Hohai University, Nanjing, People’s Republic of China; 2 School of Educational Studies, Universiti Sains Malaysia, Penang, Malaysia; 3 Department of Education, Sule Lamido University, Kafin Hausa, Nigeria; 4 College of International Languages and Cultures, Hohai University, Nanjing, People’s Republic of China; George Mason University, UNITED STATES OF AMERICA

## Abstract

**Background:**

Assessing the impact of interventions on college students’ physical activity (PA) requires the use of reliable and valid measurement tools. However, the tools employed in existing studies and their respective reliability and validity are not well-documented. This review aims to systematically evaluate the PA measurement tools utilized in interventions targeting college students and to assess the quality of their measurement properties.

**Methods:**

A comprehensive search was conducted across five databases (MEDLINE, Cochrane, Embase, Web of Science, PsycInfo) to identify studies on PA interventions among college students, using specific inclusion criteria. The screening of literature and data extraction were independently performed by two authors, focusing on the types of PA measurements used and their measurement properties.

**Results:**

A total of 52 studies, involving 63 different PA measurement tools, were included. Of these, 28 studies used self-report tools, 14 employed objective tools (with one study using two different objective tools), and 10 combined both methods. The International Physical Activity Questionnaire (IPAQ) emerged as the most frequently used self-report tool, while pedometers and accelerometers were the primary objective tools. Despite frequent references to reliability and validity, few studies provided specific evidence regarding measurement properties such as internal consistency and criterion validity, particularly those tailored to the studied population.

**Conclusion:**

The majority of PA measurement tools for college students rely on self-reported data, with limited verification of their reliability and validity. For a more accurate assessment of PA intervention effects, it is recommended to adapt the widely recognized IPAQ to specific contexts and incorporate objective tools like accelerometers, which offer practical and precise measurement within college settings.

## Introduction

The World Health Organization (WHO) has well-documented the mental and physical health benefits of physical activity (PA) across age groups [1]. For college students, adequate PA is crucial for overall health and establishing lifelong healthy habits [2]. However, participation rates remain low, with fewer than 40% meeting WHO’s PA recommendations [3,4]. An international survey spanning 23 countries found nearly half of college students engage in insufficient PA, with rates as high as 80.6% in some countries [5]. Insufficient PA among college students has become a prevalent trend, highlighting the need for effective PA interventions. While several systematic reviews and meta-analyses confirm the effectiveness of interventions using educational components, behavioral change techniques, and eHealth in improving PA among college students [6–9], the evidence, though promising, is limited by heterogeneity in PA outcomes and measurement tools, which may affect the robustness and generalizability of the findings.

Accurate, reliable, and valid tools for measuring PA are essential for tracking changes during interventions, evaluating their effectiveness, and determining the associated health benefits [10]. PA is a multifaceted behavior that includes leisure, commuting, household, and occupational activities, making comprehensive assessment challenging. Current PA measurements for college students in intervention studies predominantly rely on self-report questionnaires and objective tools. While self-report questionnaires are easy to administer and can capture a wide range of activities retrospectively, their reliability and validity are often compromised by subjective biases and limited cross-cultural applicability [11,12]. In contrast, objective tools, such as doubly labelled water (DLW), calorimetry, oxygen consumption, pedometers, and accelerometers, are considered more precise and reliable, but their use in large-scale studies is constrained by significant financial, temporal, and technical demands [13]. Regardless of the methods employed, the measurement properties of PA tools must undergo rigorous testing to ensure their reliability, validity, and practicality. Evaluating these properties typically involves assessing indicators such as reliability, validity, responsiveness, and potential biases, with careful selection of appropriate metrics tailored to the specific research context [14].

Assessment tools such as the COSMIN (Consensus-based Standards for the Selection of Health Measurement Instruments) Checklist [15] and the GAPAQ (Quality Assessment of Physical Activity Questionnaire) Checklist [16] are frequently employed to evaluate the measurement properties of PA questionnaires. Numerous studies have utilized these checklists to systematically evaluate PA questionnaires across various populations, including children and adolescents [15,17], adults [16], pregnant individuals [18], and patients [19]. Additionally, Falck et al. developed an evaluation framework to conduct a systematic review of the measurement properties of PA self-report and objective tools within elderly PA interventions [20]. These investigations provide a robust scientific foundation for PA measurement within specific cohorts.

Summarizing and evaluating the quality of PA measurement tools used in intervention studies can facilitate the selection of the most suitable tools for specific research purposes. This approach also helps mitigate biases in measurement outcomes, which is crucial for accurately interpreting the effects of interventions. Critically assessing the quality of PA measurement tools utilizing an established checklist enables the identification of their methodological rigor at both holistic and specific levels, providing a comprehensive scientific basis for their application. However, to our knowledge, there is currently no consensus on the best suitable tools for effectively and accurately measuring PA in interventions targeting college students, nor is there a systematic review addressing this issue.

Therefore, this review aims to achieve two primary objectives: first, to systematically review the application of PA measurements used in interventions targeting college students; and second, to critically evaluate and synthesize the measurement properties of these tools within the context of these interventions.

## Methods

This systematic review evaluates PA measurement tools, encompassing self-reported and objective tools, utilized in intervention studies among college students. The review adheres to the PRISMA guidelines [21], with the protocol registered on the PROSPERO platform under the registration number CRD42023486769.

### Search strategy

The search strategy employed predefined terms to explore five electronic databases: PubMed, Embase, Cochrane, Web of Science, and PsycInfo. There were no restrictions on language or publication date. The literature search was conducted on August 3, 2023, and this date served as the cut-off for including studies in this review. Following this cut-off, no further updates were made, but references from relevant reviews and primary studies were manually searched to ensure comprehensive inclusion of relevant studies. The search strategy, grounded in the framework of participants, intervention, comparator, outcomes, and study design (PICOS), focused on three primary terms: college students, physical activity, and interventions. Boolean operators were utilized to refine the search. Detailed search strategies can be found in the Supportive Information (S1 File).

### Eligibility criteria

Studies were considered eligible if they met the following criteria: (1) participants: included college students of any age capable of engaging in PA, including those who were overweight or obese, but excluding preparatory and short-term continuing education students, college staff, and individuals with significant physical disabilities or mental disorders; (2) interventions: aimed to examine PA interventions as either a primary or secondary objective, excluding studies where the purpose did not include promoting PA or exercise; (3) comparators: involved any type of control group, such as no intervention, usual care, or alternatives, with no restrictions based on the presence or type of control group; (4) outcomes: measured PA at both baseline and post-intervention to assess changes, including indicators such as energy expenditure, participation frequency, step count, duration of the activity, the intensity of activity, and metabolic equivalent tasks. Studies that did not measure changes in PA were excluded; (5) study design: comprised interventions designed to enhance PA or exercise among college students, including randomized controlled trials (RCTs), pilot RCTs, cluster-RCTs, and quasi-experimental studies. Studies that were non-peer-reviewed or unpublished theses were excluded.

### Study selection

Retrieved records were imported into EndNote 20 (Thomson ISI Research Soft, Philadelphia, PA, USA) for deduplication and initial screening based on titles and abstracts by two authors (PSY and YF), with disagreements resolved by a third author (AZK).

### Data extraction

Data from the included studies were extracted using a predefined coding scheme into a spreadsheet. The extracted data encompassed trial year and country, intervention type, mode, duration, sample size, measurement tools, outcomes, and measurement intervals. Missing data for these study characteristics were recorded as ‘Not reported’ without imputation. Two authors (PSY and YF) performed data extraction independently, with consensus reached through discussion for any discrepancies. Key measurement properties extracted included (1) citations for reliability and validity, (2) within-sample reliability, (3) criterion validity, (4) evidence of reliability and validity, and (5) population-specific measurement properties. For these properties, missing data were systematically recorded as ‘No’ (0) in the binary evaluation framework, indicating the absence of reported evidence rather than an assumption of non-existence. This ensured that evaluations were strictly based on explicitly reported study data.

### Quality assessment of measurement properties

Quality assessment of measurement properties referenced established methodologies from previous studies [16,20]. The included studies employed both self-report tools (e.g., developed or adapted questionnaires, diaries) and objective measures (e.g., DLW, calorimeters, pedometers, accelerometers, heart rate monitors, and direct observation). The assessment focused on five key measurement properties, which are further detailed below.

#### Citation for reliability and validity.

Evaluating the reliability and validity of measurement tools is fundamental to their efficacy. This study examines whether these tools were supported by citations from psychometric studies, ensuring that the reported reliability and validity are robust and applicable.

#### Within sample reliability.

This study emphasizes the importance of assessing internal consistency reliability within the specific population being studied. Ensuring that the measurement tool is reliable within the sample confirms its ability to produce stable and consistent results in the study’s context.

#### Criterion validity.

Criterion validity assesses how well a measurement tool correlates with a widely recognized standard. In PA measurement, objective tools like DLW, calorimeters, and accelerometers serve as the criterion standards. This study reviews the evidence of criterion validity provided in the included studies, focusing on the correlation between test results and established benchmarks.

#### Evidence of reliability and validity.

Reliability indicates the consistency of measurement under similar conditions, while validity refers to how accurately a tool measures the intended outcomes [22,23]. This study assesses whether the included studies explicitly reported these properties and provided evidence supporting the reliability and validity of the measurement tools.

#### Population specific measurement properties.

The study assesses the relevance of measurement properties within a specific population, considering variables like age, race, and gender. Ensuring that reliability and validity have been validated for college students is crucial for accurate interpretation of the measurement outcomes in this context [20].

The quality of measurement properties in the included studies was evaluated across five sections, encompassing eight key items. Two authors (PSY and YF) independently assessed the studies, and any discrepancies were resolved through discussion and consensus. If a consensus could not be reached, a third author (AZK) provided the final decision.

### Data analysis

Extracted study characteristics were summarized descriptively in a spreadsheet, including PA measurement adoption and their measurement properties, with binary evaluation (Yes =  1, No =  0) and aggregated statistical analysis based on quality assessment criteria, calculating percentages and mean for each entry.

## Results

### Search outcomes

**Fig 1:**
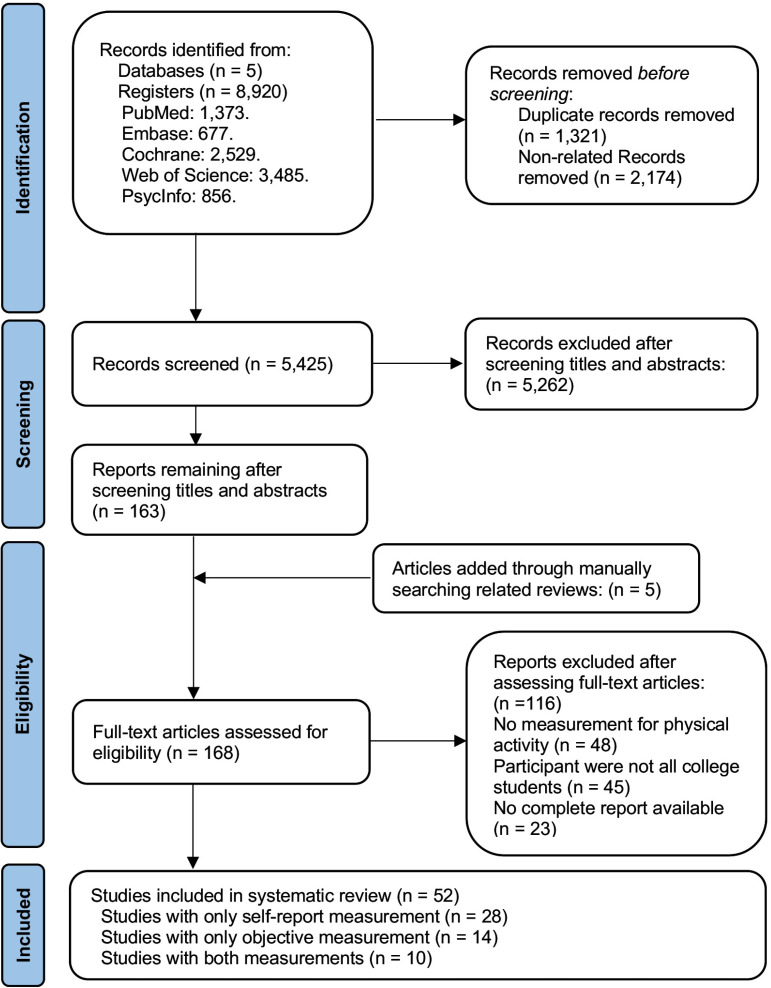
PRISMA literature searching.

The search across five databases yielded 8,920 entries. After excluding 1,321 duplicates and 2,174 unrelated records, 5,425 records underwent title and abstract screening. This process led to 163 studies being selected for full-text review. An additional five articles were added manually following a recursive search of relevant literature. According to the eligibility criteria, 116 articles were excluded based on factors including the lack of PA measurements, non-college student participants, and incomplete reports. Ultimately, 52 articles [24–75] were included in the systematic review. The literature filtering process is detailed in Fig 1.

### Characteristics of included studies

The majority of the studies (49 out of 52) were RCTs [25,26,28–38,40–75], with intervention durations ranging from 10 days to 15 months. These studies included a total of 13,761 college students, with sample sizes varying from 20 to 600 participants. The research was conducted globally: 34 studies in North America (USA: 28 [25,27,31,34–36,38,39,41–43,45,48,50–53,55,58–63,68–70,72]; Canada: 5 [40,64,71,74,75]; Mexico: 1 [67]), 12 studies in Asia (China: 6 [30,32,33,46,47,49]; Japan: 2 [37,73]; Korea [65], India [66], Jordan [24], and Malaysia [44]: 1 each), and 6 studies in Europe (UK: 2 [28,29]; Turkey: 2 [26,57]; Italy [54] and Spain [56]: 1 each). The methods for measuring PA varied among the studies, with 28 studies [24,27–29,31–33,35–40,42,45,47,48,51–53,57,63,67,69–73] using self-report tools, 13 studies [25,43,49,50,55,56,58–62,66,68] using objective tools, 10 studies [26,30,34,44,46,54,64,65,74,75] employing both methods and one study [41] using a combination of pedometers and accelerometers as objective tools. The characteristics of these studies are detailed in Table 1.

**Table 1:** Studies characteristics of included studies.

Study	Publication Year	Country	Intervention Mode	Duration	Trial Category	Sample Size	Measurement Tool	Dimension	Recall Period
Abu-Moghli et al., 2010	2010	Jordan	Health education programme	10 days	Quasi-experiment	930	Self-report items	Four behavioral category statements	NR
Al-Nawaiseh et al., 2022	2022	USA	Smartphone App	12 weeks	RCT	130	Pedometer	Steps	1 week
BarğI, 2022	2022	Turkey	PA counselling	4 weeks	RCT	31	Pedometer; The Turkish version of IPAQ-SF	Steps; PAL	1 day; 1 week
Barkley et al., 2017	2017	USA	“Poke´mon Go” Game	1 week	Quasi-experiment	358	IPAQ	EE from walks	1 week
Belogianni et al., 2023	2023	UK	Digital interventions using game-elements	10 weeks	RCT	88	IPAQ-SF	TEE	1 week
Cameron et al., 2015	2015	UK	Online theory-based intervention	1 month and 6 months	RCT	2621	IPAQ-SF	TEE	1 week
Choi et al., 2020	2020	Chinese Hong Kong	Sport education within a compulsory physical education program	10 weeks	Cluster-RCT	411	IPAQ-SF; Accelerometers	TEE; TEE	1 week
Claxton & Wells, 2009	2009	USA	PA homework	12 weeks	RCT	365	The modified questionnaire from Health People 2010	F and D for VPA, MPA, muscles, and flexibility	1 week
Duan et al., 2022	2022	China	Sequentially delivered web-based interventions	8 weeks	RCT	565	The Chinese version of IPAQ-SF	TEE	1 week
Duan et al., 2017	2017	China	Web-based intervention targeting social-cognitive indicators	8 weeks	RCT	493	The Chinese version of IPAQ-SF	TEE	1 week
Eisenberg et al., 2017	2017	USA	Electronic behavioral monitoring	1 week	RCT	146	IPAQ-SF; Accelerometer	TEE	1 week
Diez et al., 2012	2012	Mexico	Health-promoting intervention using cognitive-behavioral techniques	1 week	RCT	73	The HPLP-II-Spanish version	F	1 week
Figueroa et al., 2022	2017	USA	Daily motivational text messages	6 weeks	RCT	93	IPAQ-SF; Pedometer	TEE	1 week
Franko et al., 2008	2008	USA	Internet-based nutrition and PA education program	3 and 6 months	RCT	476	IPAQ-LF	TEE	1 week
Fukui et al., 2021	2021	Japan	“Stay-at-Home Exercise” videos	8 weeks	RCT	150	IPAQ-SF	TEE	1 week
Greene et al., 2021	2021	USA	Online healthful eating and PA program	10-lesson curriculum in 15 months	RCT	1689	IPAQ-SF	TEE	1 week
Grim et al., 2021	2021	USA	Web-based PA intervention	10 weeks	Quasi-experiment	233	7-day recall items	Measured VPA in terms of mode, duration, and day	1 week
Hall & Fong, 2003	2003	Canada	A brief time perspective intervention	3 weeks	RCT	18	The 30-day recall measure, derived from the Stanford 7-day Recall	Total time of MPA and VPA	1 week
Hojjatinia et al., 2021	2021	USA	A Digital messaging intervention	6 months	RCT	45	Accelerometer	Duration of MVPA; Step counts	1 week
Kattelmann et al., 2014	2014	USA	21 mini-educational lessons and e-mail messages (called nudges)	10 weeks	RCT	1639	IPAQ	TEE	1 week
Kim et al., 2018	2018	USA	Wearable activity tracker in a credit-based PA instructional program (PAIP)	15 weeks	RCT	187	Accelerometer	Duration	1 week
Kok et al., 2018	2018	Malaysia	Pedometer-based intervention	8 weeks	RCT	23	IPAQ-SF; Pedometer	Steps F and D, TEE	1 week
Largo-Wight et al., 2008	2008	USA	PA logs	10 weeks	RCT	136	Health Canada and National Quality Institute questions	Scores for all types of PA, leisure PA, exercise PA	1 week
Lee et al., 2012	2012	Chinese Taiwan	An intervention combining self-efficacy theory and pedometers	12 weeks	RCT	94	IPAQ; Pedometer	Steps F and D, TEE	1 week
Lin et al., 2021	2021	Chinese Taiwan	MHealth-tailored PA intervention	12 months	RCT	143	The Taiwan version of IPAQ	F and D, TEE	1 week
Loucks et al., 2021	2021	USA	Mindfulness-based program	9 weeks	RCT	96	IPAQ	F and D, TEE	1 week
Lu et al., 2023	2023	China	Tabata-style functional HIIT	12 weeks	RCT	122	Accelerometer	Freedson Adult algorithm. LPA, MPA, and VPA	1 day
Mackey et al., 2015	2015	USA	An online diet and PA program which was originally designed for use in a workplace setting	24 weeks	RCT	47	Accelerometer	TEE	3 days
Magoc et al., 2011	2011	USA	Theoretically based and web-delivered intervention	6 weeks	RCT	104	IPAQ-SF	F and D for VPA, MPA	1 week
Marenus et al., 2021	2021	USA	Aerobic and resistance training (WeActive) and mindful exercise (WeMindful) interventions	8 weeks	RCT	77	IPAQ-SF	TEE	1 week
Martens et al., 2012	2012	USA	A brief motivational intervention (a 30-minute, 1-on-1 intervention that was delivered in a MI-based framework)	One month	RCT	70	Self-report Items	F	1 week
Maselli et al., 2019	2019	Italy	Individual counselling and activity monitors	12 weeks	RCT	33	IPAQ-SF; Accelerometer	TEE;	1 week; 1 day
McDonough et al., 2022	2022	USA	A remote, YouTube-delivered exercise intervention	12 weeks	RCT	64	Accelerometer	PA intensities (i.e., MVPA, LPA, SB)	1 day
Miragall et al., 2018	2018	Spain	An internet- based motivational intervention	3 weeks	RCT	76	Pedometer	Steps	1 week
Muftuler & Ince, 2015	2015	Turkey	A PA course based on the Trans-Contextual Model	12 weeks	RCT	70	The Turkish version of IPAQ-SF	TEE	1 week
Munoz et al., 2014	2014	USA	Text messaging with pedometer intervention	10 weeks	RCT	201	Pedometer	Steps	1 day
Peng et al., 2015	2015	USA	An active video game	4 weeks	RCT	127	Accelerometer	PA intensities (i.e., MVPA, LPA, SB)	1 week
Pope et al., 2019	2019	USA	Wearable technology and social media	12 Weeks	RCT	38	Accelerometer	PA intensities (i.e., MVPA, LPA, SB)	1 week
Pope & Gao, 2022	2022	USA	A smartphone application- and social media-based intervention	10 weeks	RCT	44	Accelerometer	PA intensities (i.e., MVPA, LPA, SB)	1 week
Rote, 2017	2017	USA	A Fitbit activity monitor	1 semester	RCT	56	Pedometer	Steps	1 week
Ruissen et al., 2019	2019	Canada	Affective mental contrasting	4 weeks	RCT	110	GLTEQ; Accelerometer	PA intensities (i.e., MVPA, LPA, SB); Total and separate intensity of VPA, MPA, and LPA were scored	1 week
Schweitzer et al., 2016	2016	USA	An electronic wellness program via Email	24 weeks	Pilot RCT	148	CCPAQ	Minutes of PA	1 week
Sharp & Caperchione, 2012	2012	Canada	A pedometer-based intervention	12 weeks	RCT	184	The modified version of GLTEQ	Total and separate intensity of VPA, MPA, and LPA were scored	1 week
Shin et al., 2017	2017	Korea	An smartcare and financial incentives	12 weeks	Pilot RCT	105	The Korean version of IPAQ	TEE; Daily energy consumption (kcal)	1 week; 12 weeks
Tulasiram & Chandrasekaran, 2021	2021	India	Traditional (ACSM) and smartphone-based (SMART) exercise prescription	4 weeks	RCT	26	Pedometer	Steps	1 week
Unick et al., 2017	2017	USA	A mHealth intervention	5 weeks	RCT	61	Pedometer	Steps	1 day
Yan et al., 2023	2023	USA	An 8-week peer health coaching intervention	8 weeks	RCT	52	IPAQ-SF	TEE	1 week
Annesi et al., 2017	2017	USA	Instructional PA courses (IPACs)	10 weeks or 15 weeks	RCT	84	GLTEQ	TEE	1 week
Brown et al., 2014	2014	Canada	A residence community–based intervention	20 weeks	RCT	60	GPAQ	MVPA	Baseline; 30 days; 1 week
Heeren et al., 2018	2018	USA	Health-promotion intervention (Focus on increasing knowledge, attitudes, self-efficacy, and skills to prevent NCDs)	6 months and 12 months	RCT	176	3 open-ended items	Whether meet PA recommendations	1 week
Okazaki et al., 2014	2014	Japan	An interactive internet-based PA intervention	4 months	RCT	77	IPAQ	TEE	1 week
Sriramatr et al., 2014	2014	Canada	A Social Cognitive Theory-based internet intervention	3 months	RCT	220	The Thai Version of GLTEQ; Pedometer	TEE; Steps	1 week; 3 days

Notes: ACSM: American College of Sports Medicine; CCPAQ: The Cross-Cultural Activity Patterns Questionnaire; D: durance; EE: energy expenditure; F: frequency; GLTEQ: Godin Leisure-Time Exercise Questionnaire; GPAQ: Global Physical Activity Questionnaire; HIIT: high intensity interval training; HPLP: Health-Promoting Lifestyle Profile; IPAQ: International Physical Activity Questionnaire; LF: long form; LPA: light physical activity; MPA: moderate physical activity; MVPA: moderate to vigorous physical activity; NCD: non-communicated disease; NR: no report; PA: physical activity; PAL: physical activity level; RCT: randomized controlled trial; SB: sedentary behavior; SF: short form; SMART: smart- phone application; TEE: total energy expenditure; VPA: vigorous physical activity.

### Utilization of PA measurement tools

The 52 studies employed PA measurement tools on 63 occasions, with self-report tools used in 38 instances and objective tools in 25, as depicted in Table 2. The International Physical Activity Questionnaire (IPAQ) emerged as the most utilized self-report questionnaire [26–30,32–38,42,44,46–48,51,52,54,57,65,69,73] (24 out of 38 times), including its original English version and equivalent translations. The Godin Leisure-Time Exercise Questionnaire (GLTEQ) followed and was used in four studies [64,70,74,75]. Of the other seven self-report questionnaires [31,39,40,45,63,67,71] was each used once. Additionally, three studies [24,53,72] used self-develop items.

**Table 2:** Summary of utilization of PA measurement tools.

Measure	Frequency	Studies
**Self-report measures (n = 38)**		
International Physical Activity Questionnaire	24	[26–30,32–38,42,44,46–48,51,52,54,57,65,69,73]
The Godin Leisure-Time Exercise Questionnaire	4	[64,70,74,75]
The Cross-Cultural Activity Patterns Questionnaire	1	[50]
Global Physical Activity Questionnaire	1	[71]
Modified questionnaire from Health People 2010	1	[31]
7-day recall items	1	[39]
30-day recall measure, derived from the Stanford 7-day Recall	1	[40]
Health Canada and National Quality Institute questions	1	[45]
HPLP-II-Spanish Version	1	[67]
Unnamed Self-report items	3	[24,53,72]
**Objective measures (n = 25)**		
Pedometer	12	[25,26,41,44,46,56,58,62,64,66,68,74]
Accelerometer	13	[30,34,41,43,49,50,54,55,59–61,75,76]

Objective tools were almost evenly divided between pedometers [25,26,41,44,46,56,58,62,64,66,68,74] (12 times) and accelerometers [30,34,41,43,49,50,54,55,59–61,75,76] (13 times). A summary of utilization of PA measurements is shown in Table 2.

### Measurement properties of PA measurement tools

The detailed assessment of measurement properties for all PA measurements is presented in supporting information (shown in S1 Table). As summarized in Table 3, the measurement properties of PA measurement tools are reviewed and analyzed across the following five sections comprising eight items.

**Table 3:** Measurement properties across studies.

Measurement Property	Self-report measures (%)	Objective measures (%)	Total(%)
Citation for Reliability	49	24	73
Reliability Citation from Psychometric Study	44	21	65
Within Sample Reliability	10	1	11
Citation for Validity	47	24	71
Validity Citation from Psychometric Study	44	14	58
Criterion Based Validity	16	17	33
Reliability/Validity had been Established	19	4	23
Population-Specific Reliability and Validity	8	2	10

#### Citation for reliability and validity.

Reliability and validity were cited in 73% [26,27,29–43,46–50,52,54,56,57,59–65,67,69–71,73–75] and 71% [26,27,30–43,46–50,52,54,56,57,59–65,67,69–71,73–75] of the studies, respectively, but references from psychometric studies dropped to 65% [26,27,30,32–38,40–43,46–50,52,54,56,57,59,62–64,67,69–71,73–76] for reliability and 58% [26,27,30,32–38,40,42,46–48,50,52,54,56,57,59,62–65,67,69–71,73–75] for validity. Self-report tools citing reliability and validity in 49% [26,27,29–40,42,46–48,52,54,57,63–65,67,69–71,73–75] and 47% [26,27,30–40,42,46–48,52,54,57,63–65,67,69–71,73–75] of cases, respectively, with psychometric studies cited in 44% for both reliability [26,27,30,32–38,40,42,46–48,52,57,63–65,67,69–71,73–75] and validity [26,27,30,32–38,40,42,46–48,52,54,57,62–65,67,69–71,73–75]. Objective tools showed a 24% reference rate for both reliability [26,30,34,41,43,49,50,54,56,59–62,75] and validity [26,30,34,41,43,49,50,54,56,59–62,75], with 21% [26,30,34,41,43,49,50,54,56,59,62,75] citations of psychometric studies for reliability and 14% [26,30,34,50,54,56,59,62,75] for validity.

#### Within sample reliability.

Only seven studies [24,31,36,52,64,66,67] (11%) tested reliability within their samples, predominantly self-report tools, with a single study [66] using objective tools.

#### Criterion validity evidence.

Evidence of criterion validity was evenly split between self-report [27,34,36,39,52,54,57,64,70,75], and objective tools [26,34,43,50,54,56,59,61,62,66,75] across 21 studies.

#### Explicit reporting of reliability and validity.

Twelve self-report tools [26,27,30,34,36,39,47,52,57,67,70,75] and three objective tools [26,30,34] detailed explicit reliability and validity parameters, with four studies [52,57,67,70] focusing solely on internal consistency.

#### Population-specific evidence.

Evidence specific to college students’ reliability and validity was found in only six studies [46,47,52,64,66,67], five of which were self-report tools [46,47,52,64,67], and one [66] used an objective tool. One study [24] only described the evidence of reliability.

## Discussion

This review evaluated PA measurement tools used in college student interventions, revealing that self-report methods, particularly the IPAQ and GLTEQ, are the most common, with some studies using unspecified self-report items. Objective tools, like pedometers and accelerometers, are also frequently employed. While many interventions reference the reliability and validity of these tools, only a small percentage provide detailed evidence for key measurement properties such as internal consistency, criterion validity, and population-specific reliability. This underscores the need for more rigorous evaluation and reporting to enhance the accuracy and applicability of these measurement tools.

The self-report measures of PA involve participants documenting or recalling their activities (including mode, intensity, frequency, duration, times, intervals, etc.) over a specified period [77]. Due to their ease of operation and comprehensive coverage of various PA, self-report questionnaires are predominantly used in measuring adults’ PA [12]. This review further validated their extensive adoption in PA intervention studies of college students.

The IPAQ, developed by a multinational working group, assesses PA behaviors by recalling activities of varying intensities over the past week [10]. It is widely used among adults aged 15 to 59 years [78]. The IPAQ is available in two forms: the short form that evaluates the duration of vigorous, moderate, walking, and sedentary behaviors through 7 items, and the long form that collects data on activities related to housework, commuting, occupation, leisure, and sedentary behaviors through 27 items [78]. This review primarily found the short form in use, with only one study employing the long form. Previous studies have demonstrated IPAQ’s good reliability and validity across diverse populations, including adolescents and adults [79,80]. Ding et al. [81] evaluated the short form’s psychometric properties in college students, revealing high reliability (ICC =  0.71 - 0.89) and criterion validity comparable to other questionnaires (correlation coefficients with accelerometers and pedometers ranged from 0.15 to 0.26). The measurement properties of IPAQ’s various language versions have been extensively validated [78], though some variability in reliability and validity has been noted, leading to debates about its measurement efficiency [79]. Most studies included in this review cited IPAQ’s reliability and validity, but only two studies tested its reliability within college student samples [36,52]. Five studies reported the criterion validity of IPAQ measurements with accelerometers [27,34,36,52,57], and three discussed its measurement properties specifically in college students [46,47,52]. These findings support the use of IPAQ in intervention research, though more precise validation in this population is needed. While evidence suggests high reliability, criterion validity is modest. The accuracy of self-report measures compared to objective methods remains debated, underscoring the need for ongoing refinement of these tools. The IPAQ-A, a version modified for adolescents, showed satisfactory criterion validity [10], suggesting potential strategies for improving the IPAQ’s application in college students.

The GLTEQ is another retrospective self-report questionnaire that assesses the frequency of engaging in activities of three different intensities for more than 15 minutes over the past week [82]. The total leisure activity is calculated by multiplying the frequency of these activities by their respective intensity metabolic scores. In this review, four studies utilized the GLTEQ [64,70,74,75], with two providing evidence of its reliability and validity [64,75]. Due to the arbitrary nature of the 15-minute activity duration, two studies adjusted this criterion [64,74]. While the GLTEQ effectively measures PA, its focus solely on leisure-time activities limits its applicability, as it omits other PA forms.

Other self-report questionnaires were used less frequently, with the Global Physical Activity Questionnaire (GPAQ) being a notable example. Developed by the WHO as a revision of the IPAQ, the latest version of GPAQ includes 16 items that investigate occupational, transportation, and leisure activities [83]. Reliability and validity assessments in adults across nine countries have shown moderate to strong reliability and validity levels comparable to the IPAQ [84]. However, its criterion validity, particularly when compared to objective measurements, is weaker.

Objective tools for measuring PA include direct observation, DLW, calorimeters, heart rate monitors, accelerometers, and pedometers [13]. These tools, free from subjective biases, are valued for their precision. While there is no globally accepted gold standard for PA measurement, objective tools often serve as benchmarks for validating self-report measures due to their recognized accuracy and reliability [85]. However, these tools are not without limitations. For example, DLW measures total energy expenditure without distinguishing between activity types, and its complex, costly protocol is impractical for large-scale use. Accelerometers, although convenient, may underestimate PA due to algorithmic constraints, and pedometers, while accurate in counting steps, do not assess activity intensity.

In this review, only accelerometers and pedometers were identified as objective tools. Their adherence to measurement property checklists is generally lower than that of self-report tools. A recent study evaluated the reliability of pedometers [66], but the practice of validating the in-sample reliability of objective tools remains uncommon. This lack of validation raises concerns about the integrity of objectively measured PA data. Additionally, while some studies cite evidence of reliability and validity, the specific reliability and validity of objective tools in college students are rarely examined [86]. This gap could introduce significant random and systematic errors, potentially distorting true outcomes.

To ensure reliability and validity in PA intervention studies among college students, it is essential to prioritize objective tools specifically validated for this population, particularly those that ensure both accurate and reliable measurement. Despite the inherent limitations of various objective measurements, advancements in pattern recognition and machine learning integrated with accelerometers offer promising improvements [87]. However, these technologies require extensive validation across diverse populations to ensure their broad applicability.

The use of accurate and reliable measures is crucial for successful PA interventions [77]. Given the superior accuracy of objective tools, their use is highly recommended when feasible. College students, who typically have access to consistent environments and health resources, benefit from the implementation of these tools with the support of educational and health professionals [88]. Pedometers provide a simple method for estimating general activity levels, while accelerometers are better suited for detailed assessments of activity intensity and duration. These tools must meet established reliability and validity standards within the target population.

In cases where objective measures are not feasible for large samples, self-report questionnaires are an alternative. However, researchers should avoid using custom, unvalidated items due to potential biases and compromised data integrity. The IPAQ, despite not being flawless, is widely recognized for its measurement efficacy in numerous studies [10,79,89]. When aligned with study objectives, the IPAQ can produce reliable outcomes. Adapting it for college students offers a promising avenue for future research.

This systematic review categorizes and synthesizes PA measurement tools used in interventions targeting college students, providing a comprehensive evaluation of their measurement properties. While the study significantly contributes to the evidence base for PA measurement in this area, several limitations should be noted. First, despite a comprehensive search strategy, some studies may have been inadvertently missed. Second, the review assessed measurement properties using eight criteria, but the critical metric of responsiveness was omitted due to a lack of reports in the included studies. Responsiveness, essential for evaluating a tool’s ability to detect changes post-intervention, was thus underrepresented, impacting the scientific rigor of the evaluation. Third, the binary (yes or no) approach used for assessing measurement properties lacked standardized criteria, limiting the nuanced quantification of individual study quality. Finally, the interventions reviewed were primarily tested on college students in specific regions, limiting the generalizability of the findings. Broader validation across different populations and regions is necessary to ensure the applicability of the measurement tools globally.

## Conclusion

This review investigated the use of PA measurement tools in interventions targeting college students, offering a comprehensive evaluation of their measurement properties. The findings highlight the need for rigorous psychometric validation of PA measurement tools in this demographic, emphasizing the importance of selecting tools that are both reliable and valid. Future research should focus on adapting widely used questionnaires, such as the IPAQ, to better address the specific characteristics of college students. Additionally, integrating objective tools like accelerometers and exploring advanced technologies can improve the precision and scientific rigor of PA measurement, offering promising directions for enhancing the assessment of PA interventions.

## Supporting information

S1 File Search strategy. (DOCX)

S2 File Literature screening procedure. (XLSX)

S1 Table Assessment of measurement properties. (DOCX)

S2 Table Extracted data and eligibility confirmation. (XLSX)

S1 Checklist PRISMA_2020_checklist. (DOCX)

## References

[1] World Health Organization. WHO guidelines on physical activity and sedentary behaviour, 2020. Geneva: World Health Organization; 2020. Licence: CC BY-NC-SA.

[2] Maselli M, Ward PB, Gobbi E, Carraro A. Promoting physical activity among university students: A systematic review of controlled trials. Am J Health Promot. 2018;32(7):1602–12. doi: 10.1177/0890117117753798 29366334

[3] Keating XD, Guan J, Piñero JC, Bridges DM. A meta-analysis of college students’ physical activity behaviors. J Am Coll Health. 2005;54(2):116–125. doi: 10.3200/JACH.54.2.116-126 16255324

[4] Pengpid S, Peltzer K. Sedentary behaviour, physical activity and life satisfaction, happiness and perceived health status in university students from 24 countries. Int J Environ Res Public Health. 2019;16(12):2084. doi: 10.3390/ijerph16122084 31200427 PMC6617209

[5] Pengpid S, Peltzer K, Kassean HK, Tsala Tsala JP, Sychareun V, Muller-Riemenschneider F. Physical inactivity and associated factors among university students in 23 low-, middle- and high-income countries. Int J Public Health. 2015;60(5):539–549. doi: 10.1007/s00038-015-0680-0 25926342

[6] Whatnall MC, Sharkey T, Hutchesson MJ, Haslam RL, Bezzina A, Collins CE, et al. Effectiveness of interventions and behaviour change techniques for improving physical activity in young adults: A systematic review and meta-analysis. J Sports Sci. 2021;39(15):1754–1771. doi: 10.1080/02640414.2021.1898107 33685357

[7] Peng S, Yuan F, Othman AT, Zhou X, Shen G, Liang J. The Effectiveness of E-Health Interventions Promoting Physical Activity and Reducing Sedentary Behavior in College Students: A Systematic Review and Meta-Analysis of Randomized Controlled Trials. Int J Environ Res Public Health. 2022;20(1):318. doi: 10.3390/ijerph20010318 PMID: 36612643 10.3390/ijerph20010318 PMC9819541

[8] Maselli M, Ward PB, Gobbi E, Carraro A. Promoting physical activity among university students: A systematic review of controlled trials. Am J Health Promot. 2018;32(7):1602–12. doi: 10.1177/0890117117753798 29366334

[9] Peng SY, Othman AT, Khairani AZ, Zeng G, Zhou XG, Fang Y. Effectiveness of pedometer- and accelerometer-based interventions in improving physical activity and health-related outcomes among college students: A systematic review and meta-analysis. Digit Health. 2023;9:20552076231188213. doi: 10.1177/20552076231188213 37492032 PMC10364418

[10] Rachele JN, McPhail SM, Washington TL, Cuddihy TF. Practical physical activity measurement in youth: A review of contemporary approaches. World J Pediatr. 2012;8(3):207–16. doi: 10.1007/s12519-012-0359-z 22886192

[11] Prince SA, Cardilli L, Reed JL, Saunders TJ, Kite C, Douillette K, et al. A comparison of self-reported and device-measured sedentary behaviour in adults: A systematic review and meta-analysis. Int J Behav Nutr Phys Act. 2020;17(1):31. doi: 10.1186/s12966-020-00938-3 32131845 PMC7055033

[12] Dowd KP, Szeklicki R, Minetto MA, Murphy MH, Polito A, Ghigo E, et al. A systematic literature review of reviews on techniques for physical activity measurement in adults: A DEDIPAC study. Int J Behav Nutr Phys Act. 2018;15(1):15. doi: 10.1186/s12966-017-0636-2 29422051 PMC5806271

[13] Hills AP, Mokhtar N, Byrne NM. Assessment of physical activity and energy expenditure: An overview of objective measures. Front Nutr. 2014;1:5. doi: 10.3389/fnut.2014.00005 25988109 PMC4428382

[14] Lee EH, Kang EH, Kang HJ. Evaluation of Studies on the Measurement Properties of Self-Reported Instruments. Asian Nurs Res (Korean Soc Nurs Sci). 2020;14(5):267–76. doi: 10.1016/j.anr.2020.11.004 33279657

[15] Mokkink LB, Terwee CB, Patrick DL, Alonso J, Stratford PW, Knol DL, et al. The COSMIN checklist for assessing the methodological quality of studies on measurement properties of health status measurement instruments: An international Delphi study. Qual Life Res. 2010;19(4):539–49. doi: 10.1007/s11136-010-9606-8 20169472 PMC2852520

[16] van Poppel MN, Chinapaw MJ, Mokkink LB, van Mechelen W, Terwee CB. Physical activity questionnaires for adults: A systematic review of measurement properties. Sports Med. 2010;40(7):565–600. doi: 10.2165/11531930-000000000-00000 20545381

[17] Poitras VJ, Gray CE, Borghese MM, Carson V, Chaput J-P, Janssen I, et al. Systematic review of the relationships between objectively measured physical activity and health indicators in school-aged children and youth. Appl Physiol Nutr Metab. 2016;41(6 Suppl 3):S197–239. doi: 10.1139/apnm-2015-0663 27306431

[18] Sattler MC, Jaunig J, Watson ED, van Poppel MN, Mokkink LB, Terwee CB, et al. Physical activity questionnaires for pregnancy: A systematic review of measurement properties. Sports Med. 2018;48(10):2317–46. doi: 10.1007/s40279-018-0961-x 30094797 PMC6132497

[19] Martins JC, Aguiar LT, Nadeau S, Scianni AA, Teixeira-Salmela LF, Faria C. Measurement properties of self-report physical activity assessment tools for patients with stroke: A systematic review. Braz J Phys Ther. 2019;23(6):476–90. doi: 10.1016/j.bjpt.2019.02.004 30872006 PMC6849082

[20] Falck RS, McDonald SM, Beets MW, Brazendale K, Liu-Ambrose T. Measurement of physical activity in older adult interventions: A systematic review. Br J Sports Med. 2016;50(8):464–70. doi: 10.1136/bjsports-2014-094413 26276362

[21] Page MJ, McKenzie JE, Bossuyt PM, Boutron I, Hoffmann TC, Mulrow CD, et al. The PRISMA 2020 statement: An updated guideline for reporting systematic reviews. Int J Surg. 2021;88:105906. doi: 10.1016/j.ijsu.2021.105906 33789826

[22] Kimberlin CL, Winterstein AG. Validity and reliability of measurement instruments used in research. Am J Health Syst Pharm. 2008;65(23):2276–84. doi: 10.2146/ajhp070364 19020196

[23] Heale R, Twycross A. Validity and reliability in quantitative studies. Evid Based Nurs. 2015;18(3):66–7. doi: 10.1136/eb-2015-102129 25979629

[24] Abu-Moghli FA, Khalaf IA, Barghoti FF. The influence of a health education programme on healthy lifestyles and practices among university students. Int J Nurs Pract. 2010;16(1):35–42. doi: 10.1111/j.1440-172X.2009.01801.x 20158546

[25] Al-Nawaiseh HK, McIntosh WA, McKyer LJ. An-m-Health intervention using smartphone app to improve physical activity in college students: A randomized controlled trial. Int J Environ Res Public Health. 2022;19(12):7228. doi: 10.3390/ijerph19127228 35742477 PMC9223541

[26] BarĞI G. Effectiveness of physical activity counseling in university students educated by distance learning during COVID-19 pandemic: A randomized-controlled trial. J Basic Clin Health Sci. 2022;6(2):374–84. doi: 10.30621/jbachs.1027410 36421083

[27] Barkley JE, Lepp A, Glickman EL. “Pokemon go!” may promote walking, discourage sedentary behavior in college students. Games Health J. 2017;6(3):165–70. doi: 10.1089/g4h.2017.0009 28628384

[28] Belogianni K, Ooms A, Lykou A, Nikoletou D, Jayne Moir H. An online game-based intervention using quizzes to improve nutrition and physical activity outcomes among university students. Health Education Journal. 2023;82(6):636–50. doi: 10.1177/00178969231179032

[29] Cameron D, Epton T, Norman P, Harris PR, Webb TL, Sheeran P. A theory-based online health behaviour intervention for new university students (U@Uni): results from a repeat randomized controlled trial. Trials. 2015;16:555. doi: 10.1186/s13063-015-1092-4 26643917 PMC4672536

[30] Choi SM, Sum KWR, Leung FLE, Ng GYF, Tsang WWN, Tse MA, et al. Effect of sport education on students’ perceived physical literacy, motivation, and physical activity levels in university required physical education: A cluster-randomized trial. High Educ. 2020;81(6):1137–55. doi: 10.1007/s10734-020-00603-5

[31] Claxton D, Wells GM. The effect of physical activity homework on physical activity among college students. J Phys Act Health. 2009;6(2):203–10. doi: 10.1123/jpah.6.2.203 19420398

[32] Duan Y, Liang W, Wang Y, et al. The effectiveness of sequentially delivered web-based interventions on promoting physical activity and fruit-vegetable consumption among chinese college students: mixed methods study. J Med Internet Res. 2022;24(1). doi: 10.2196/30566 35080497 PMC8829698

[33] Duan YP, Wienert J, Hu C, Si GY, Lippke S. Web-based intervention for physical activity and fruit and vegetable intake among chinese university students: A randomized controlled trial. J Med Internet Res. 2017;19(4). doi: 10.2196/jmir.7152 28396306 PMC5404143

[34] Eisenberg MH, Phillips LA, Fowler L, Moore PJ. The impact of e-diaries and accelerometers on young adults’ perceived and objectively assessed physical activity. Psychol Sport Exerc. 2017;30:55–63. doi: 10.1016/j.psychsport.2017.01.008 28966555 PMC5619258

[35] Figueroa CA, Deliu N, Chakraborty B, et al. Daily motivational text messages to promote physical activity in university students: results from a microrandomized trial. Ann Behav Med. 2022;56(2):212–18. doi: 10.1093/abm/kaab028 33871015

[36] Franko DL, Cousineau TM, Trant M, Green TC, Rancourt D, Thompson D, et al. Motivation, self-efficacy, physical activity and nutrition in college students: randomized controlled trial of an internet-based education program. Prev Med. 2008;47(4):369–77. doi: 10.1016/j.ypmed.2008.06.013 18639581 PMC2926661

[37] Fukui K, Suzuki Y, Kaneda K, Koizumi Y, Nakamura T, Ishikawa A, et al. Do “stay-at-home exercise” videos induce behavioral changes in college students? a randomized controlled trial. Sustainability. 2021;13(21):11600. doi: 10.3390/su132111600

[38] Greene GW, White AA, Hoerr SL, Lohse B, Schembre SM, Riebe D, et al. Impact of an online healthful eating and physical activity program for college students. Am J Health Promot. 2012;27(2). doi: 10.4278/ajhp.110606-QUAN-239 23113786

[39] Grim M, Hortz B, Petosa R. Impact evaluation of a pilot web-based intervention to increase physical activity. Am J Health Promot. 2011;25(4):227–230. doi: 10.4278/ajhp.081216-ARB-307 21361806

[40] Hall PA, Fong GT. The effects of a brief time perspective intervention for increasing physical activity among young adults. Psychol Health. 2003;18(6):685–706. doi: 10.1080/0887044031000110447

[41] Hojjatinia S, Hojjatinia S, Lagoa CM, Brunke-Reese D, Conroy DE. Person-specific dose-finding for a digital messaging intervention to promote physical activity. Health Psychol. 2021;40(8):502–512. doi: 10.1037/hea0001117 34618498 PMC8514055

[42] Kattelmann KK, Bredbenner CB, White AA, Greene GW, Hoerr SL, Kidd T, et al. The effects of young adults eating and active for health (YEAH): A theory-based Web-delivered intervention. J Nutr Educ Behav. 2014;46(6). doi: 10.1016/j.jneb.2014.08.007 25457733

[43] Kim Y, Lumpkin A, Lochbaum M, Stegemeier S, Kitten K. Promoting physical activity using a wearable activity tracker in college students: A cluster randomized controlled trial. J Sports Sci. 2018;36(16):1889–96. doi: 10.1080/02640414.2018.1423886 29318916

[44] Kok JL, Asma A, Khairil-Shazmin K, Hayati MY. A Pedometer-based intervention with daily walking steps and its relationship with nutritional status among overweight/Obese University Students in Kuala Terengganu. Int Med J Malaysia. 2018;17(3):17–27.

[45] Largo-Wight E, Todorovich JR, O’Hara BK. Effectiveness of point-based physical activity intervention. Phys Educ. 2008;65(1):30–45.

[46] Lee LL, Kuo YC, Fanaw D, Perng SJ, Juang IF. The effect of an intervention combining self-efficacy theory and pedometers on promoting physical activity among adolescents. J Clin Nurs. 2012;21(7–8):914–22. doi: 10.1111/j.1365-2702.2011.03881.x 22082301

[47] Lin PJ, Fanjiang YY, Wang JK, Wu MH, Liu C, Wu FF, et al. Long-term effectiveness of an mHealth-tailored physical activity intervention in youth with congenital heart disease: A randomized controlled trial. J Adv Nurs. 2021;77(8):3494–3506. doi: 10.1111/jan.14924 34151444

[48] Loucks EB, Nardi WR, Gutman R, Kronish I, Saadeh FB, Li Y, et al. Mindfulness-based college: A stage 1 randomized controlled trial for university student well-being. Psychosom Med. 2021;83(6):602–14. doi: 10.1097/PSY.0000000000000860 32947581 PMC8257475

[49] Lu Y, Wiltshire HD, Baker JS, Wang Q, Ying S. The effect of Tabata-style functional high-intensity interval training on cardiometabolic health and physical activity in female university students. Front Physiol. 2023;14:1095315. doi: 10.3389/fphys.2023.1095315 36923290 PMC10008870

[50] Mackey E, Schweitzer A, Hurtado ME, Ebbeling CB, Rodriguez NR, Handy LH, et al. The feasibility of an e-mail-delivered intervention to improve nutrition and physical activity behaviors in African American college students. J Am Coll Health. 2015;63(2):109–17. doi: 10.1080/07448481.2014.990971 25611932 PMC4334675

[51] Magoc D, Tomaka J, Bridges-Arzaga A. Using the web to increase physical activity in college students. Am J Health Behav. 2011;35(2):142–154. doi: 10.5993/AJHB.35.2.2 21204677

[52] Marenus MW, Murray A, Friedman K, Cunningham T, Waligora A, Smaldone A. Feasibility and effectiveness of the Web-Based we active and we mindful interventions on physical activity and psychological well-being. Biomed Res Int. 2021;2021:8400241. doi: 10.1155/2021/8400241 34660800 PMC8519690

[53] Martens MP, Buscemi J, Smith AE, Murphy JG. The short-term efficacy of a brief motivational intervention designed to increase physical activity among college students. J Phys Act Health. 2012;9(4):525–532. doi: 10.1123/jpah.9.4.525 21934167

[54] Maselli M, Gobbi E, Carraro A. Effectiveness of individual counseling and activity monitors to promote physical activity among university students. J Sports Med Phys Fitness. 2019;59(1):132–140. doi: 10.23736/S0022-4707.17.07981-6 29199784

[55] McDonough DJ, Helgeson MA, Liu W, Gao Z. Effects of a remote, YouTube-delivered exercise intervention on young adults’ physical activity, sedentary behavior, and sleep during the COVID-19 pandemic: Randomized controlled trial. J Sport Health Sci. 2022;11(2):145–156. doi: 10.1016/j.jshs.2021.07.009 34314877 PMC8487769

[56] Miragall M, Dominguez-Rodriguez A, Navarro J, Cebolla A, Banos RM. Increasing physical activity through an Internet-based motivational intervention supported by pedometers in a sample of sedentary students: A randomised controlled trial. Psychol Health. 2018;33(4):465–482. doi: 10.1080/08870446.2017.1368511 28880576

[57] Muftuler M, Ince ML. Use of trans-contextual model-based physical activity course in developing leisure-time physical activity behavior of university students. Percept Mot Skills. 2015;121(1):31–55. doi: 10.2466/06.PMS.121c13x1 26226283

[58] Munoz LR, La France K, Dominguez D, et al. Text messaging as a tool to increase physical activity in college students. Phys Educ. 2014;71(3):442–58.

[59] Peng W, Pfeiffer KA, Winn B, Lin JH, Sutton D. A pilot randomized, controlled trial of an active video game physical activity intervention. Health Psychol. 2015;34S:1229–39. doi: 10.1037/hea0000302 26651464

[60] Pope ZC, Barr-Anderson DJ, Lewis BA, Pereira MA, Gao Z. Use of wearable technology and social media to improve physical activity and dietary behaviors among college students: A 12-week randomized pilot study. Int J Environ Res Public Health. 2019;16(19):3579. doi: 10.3390/ijerph16193579 31557812 PMC6801802

[61] Pope ZC, Gao Z. Feasibility of smartphone application- and social media-based intervention on college students’ health outcomes: A pilot randomized trial. J Am Coll Health. 2022;70(1):89–98. doi: 10.1080/07448481.2020.1726925 32150514

[62] Rote AE. Physical activity intervention using Fitbits in an introductory college health course. Health Educ J. 2016;76(3):337–48. doi: 10.1177/0017896916674505 28989489

[63] Schweitzer AL, Ross JT, Klein CJ, Lei KY, Mackey ER. An Electronic wellness program to improve diet and exercise in college students: A pilot study. JMIR Res Protoc. 2016;5(1):e29. doi: 10.2196/resprot.4855 26929118 PMC4791526

[64] Sharp P, Caperchione C. The effects of a pedometer-based intervention on first-year university students: A randomized control trial. J Am Coll Health. 2016;64(8):630–38. doi: 10.1080/07448481.2016.1217538 27471879

[65] Shin DW, Yun JM, Shin JH, Kwon H, Min HY, Joh HK, et al. Enhancing physical activity and reducing obesity through smartcare and financial incentives: A pilot randomized trial. Obesity. 2017;25(2):302–10. doi: 10.1002/oby.21731 28063226

[66] Tulasiram B, Chandrasekaran B. Are Smartphones better in guiding physical activity among sedentary young adults? a randomised controlled trial. Muscles Ligaments Tendons J. 2021;11(01):83–91. doi: 10.32098/mltj.01.2021.10

[67] Ulla Diez SM, Fortis AP, Franco SF. Efficacy of a health-promotion intervention for college students: A randomized controlled trial. Nurs Res. 2012;61(2):121–132. doi: 10.1097/NNR.0b013e3182475aaa 22307144

[68] Unick JL, Lang W, Williams SE, Bond DS, Egan CM, Espeland MA, et al. Objectively-assessed physical activity and weight change in young adults: A randomized controlled trial. Int J Behav Nutr Phys Act. 2017;14(1):165. doi: 10.1186/s12966-017-0620-x 29202850 PMC5715643

[69] Yan Z, Peacock J, Cohen JFW, Li Z, Maron L, Berkowitz SA, et al. An 8-week peer health coaching intervention among college students: A pilot randomized study. Nutrients. 2023;15(5):1284. doi: 10.3390/nu15051284 36904282 PMC10005245

[70] Annesi JJ, Porter KJ, Hill GM, Goldfine BD. Effects of instructional physical activity courses on overall physical activity and mood in university students. Res Q Exerc Sport. 2017;88(3):358–364. doi: 10.1080/02701367.2017.1336280 28636503

[71] Brown DM, Bray SR, Beatty KR, Kwan MY. Healthy active living: A residence community–based intervention to increase physical activity and healthy eating during the transition to first-year university. J Am Coll Health. 2014;62(4):234–242. doi: 10.1080/07448481.2014.887572 24499161

[72] Heeren GA, Jemmott JB, Marange CS, Tyler JC, Ngwane Z, Mandeya A, et al. Health-promotion intervention increases self-reported physical activity in Sub-Saharan African University Students: A randomized controlled pilot study. Behav Med. 2018;44(4):297–305. doi: 10.1080/08964289.2017.1350134 28682186 PMC6292207

[73] Okazaki K, Okano S, Haga S, Seki A, Suzuki H, Takahashi K. One-year outcome of an interactive internet-based physical activity intervention among university students. Int J Med Inform. 2014;83(5):354–360. doi: 10.1016/j.ijmedinf.2014.01.012 24636701

[74] Sriramatr S, Berry TR, Spence JC. An Internet-based intervention for promoting and maintaining physical activity: A randomized controlled trial. Am J Health Behav. 2014;38(3):430–39. doi: 10.5993/AJHB.38.3.12 25181763

[75] Ruissen GR, Rhodes RE, Crocker PRE, Beauchamp MR. Affective mental contrasting to enhance physical activity: A randomized controlled trial. Health Psychol. 2018;37(1):51–60. doi: 10.1037/hea0000551 28981303

[76] Shin DW, Joh HK, Yun JM, Kwon H, Min HY, Shin JH, et al. Design and baseline characteristics of participants in the Enhancing Physical Activity and Reducing Obesity through Smartcare and Financial Incentives (EPAROSFI): A pilot randomized controlled trial. Contemp Clin Trials. 2016;47:115–122. doi: 10.1016/j.cct.2015.12.019 26744232

[77] Sylvia LG, Bernstein EE, Hubbard JL, Keating L, Anderson EJ. A practical guide to measuring physical activity. J Acad Nutr Diet. 2014;114(2):199–208. doi: 10.1016/j.jand.2013.09.018 24290836 PMC3915355

[78] Craig CL, Marshall AL, Sjöström M, Bauman AE, Booth ML, Ainsworth BE, et al. International physical activity questionnaire: 12-country reliability and validity. Med Sci Sports Exerc. 2003;35(8):1381–95. doi: 10.1249/01.MSS.0000078924.61453.FB 12900694

[79] Sember V, Meh K, Soric M, Starc G, Rocha P, Jurak G, et al. Validity and reliability of international physical activity questionnaires for adults across EU countries: systematic review and meta analysis. Int J Environ Res Public Health. 2020;17(19):7161. doi: 10.3390/ijerph17197161 33007880 PMC7579664

[80] Guedes DP, Lopes CC, Guedes JERP. Reprodutibilidade e validade do Questionário Internacional de Atividade Física em adolescentes. Rev Bras Med Esporte. 2005;11(2):151–8. doi: 10.1590/s1517-86922005000200011

[81] Dinger MK, Behrens TK, Han JL. Validity and reliability of the international physical activity questionnaire in college students. Am J Health Educ. 2006;37(6):337–43. doi: 10.1080/19325037.2006.10598924

[82] Godin G, Shephard RJ. Leisure Time Exercise Questionnaire. Can J Appl Sport Sci. 1985;10(1):141–6. 4053261

[83] Keating XD, Zhou K, Liu X, Hodges M, Liu J, Guan J. Reliability and concurrent validity of global physical activity questionnaire (GPAQ): A systematic review. Int J Environ Res Public Health. 2019;16(21):4128. doi: 10.3390/ijerph16214128 31717742 PMC6862218

[84] Bull FC, Maslin TS, Armstrong T. Global physical activity questionnaire (GPAQ): nine country reliability and validity study. J Phys Act Health. 2009;6(6):790–804. doi: 10.1123/jpah.6.6.790 20101923

[85] Migueles JH, Cadenas-Sanchez C, Ekelund U, Delisle Nyström C, Mora-Gonzalez J, Löf M, et al. Accelerometer data collection and processing criteria to assess physical activity and other outcomes: A systematic review and practical considerations. Sports Med. 2017;47(9):1821–45. doi: 10.1007/s40279-017-0716-0 28303543 PMC6231536

[86] Downs A, Van Hoomissen J, Lafrenz A, Julka DL. Accelerometer-measured versus self-reported physical activity in college students: implications for research and practice. J Am Coll Health. 2014;62(3):204–12. doi: 10.1080/07448481.2013.877018 24377672

[87] Clark CCT, Barnes CM, Stratton G, McNarry MA, Mackintosh KA, Summers HD. A Review of Emerging Analytical Techniques for Objective Physical Activity Measurement in Humans. Sports Med. 2017;47(3):439–447. doi: 10.1007/s40279-016-0585-y 27402456

[88] Islamov IA. Fundamentals of promotion of sports and competitions and physical training among school students. Curr Res J Pedagog. 2021;2(6):85–9. doi: 10.37547/pedagogy-crjp-02-06-17

[89] Murphy JJ, Murphy MH, MacDonncha C, Murphy N, Nevill AM, Woods CB. Validity and reliability of three self-report instruments for assessing attainment of physical activity guidelines in university students. Meas Phys Educ Exerc Sci. 2017;21(3):134–41. doi: 10.1080/1091367X.2017.1297110

