# Supplementary material for: Physical activity measurement tools among college students in intervention studies: A systematic review
Source: PLoS One. 2025 Apr 10;20(4):e0321593. doi: 10.1371/journal.pone.0321593 (PMC11984739; doi:10.1371/journal.pone.0321593)
Supplement: S1 File — (DOCX) [file pone.0321593.s001.docx]

**Search strategies**

Pubmed:

((((((intervention*[Title/Abstract]) OR (trial*[Title/Abstract])) OR (experiment*[Title/Abstract])) OR (promot*[Title/Abstract])) OR (increas*[Title/Abstract])) AND (((university student*[Title/Abstract]) OR (college student*[Title/Abstract])) OR (young adul*[Title/Abstract]))) AND (("Exercise"[Mesh]) OR ((physical activity[Title/Abstract]) OR (exercise[Title/Abstract])))

Embase:

Session Results

No. Query Results

#1: 'physical activity'/exp

#2: 'physical activity':ti,ab OR 'exercise':ab,ti

#3: 'college student*':ti,ab OR 'university student*':ab,ti OR 'young adult*':ab,ti

#4: 'intervention':ti,ab OR 'trial':ab,ti OR 'experiment':ab,ti OR 'promot*':ab,ti OR 'increas*':ab,ti

#5: #1 OR #2

#6: #3 AND #4 AND #5

Cochrane Libarary:

#1: MeSH descriptor: [Exercise] explode all trees

#2: (physical activity):ti,ab,kw OR (exercise):ti,ab,kw

#3: #1 OR #2

#4: (intervention*):ti,ab,kw OR (trial*):ti,ab,kw OR (experiment*):ti,ab,kw OR (promot*):ti,ab,kw OR (increase*):ti,ab,kw

#5: (university student*):ti,ab,kw OR (college student*):ti,ab,kw OR (young adult*):ti,ab,kw

#6: #3 AND #4 AND #5

Web of Science:

#1: intervention* (Abstract) OR trial* (Abstract) OR experiment* (Abstract) OR promot* (Abstract) OR increas* (Abstract)

#2: physical activity (Abstract) OR exercis* (Abstract)

#3: university student* (Abstract) OR college student* (Abstract) OR young adult* (Abstract)

#4: #1 AND #2 AND #3

PsycInfo:

AB (physical activity or exercise) AND AB (intervention* or trial* or experiment* or promot* or increase* ) AND AB ( university student* or college student* or young adult* )
