# Supplementary material for: Physical activity measurement tools among college students in intervention studies: A systematic review
Source: PLoS One. 2025 Apr 10;20(4):e0321593. doi: 10.1371/journal.pone.0321593 (PMC11984739; doi:10.1371/journal.pone.0321593)
Supplement: S1 Table — (DOCX) [file pone.0321593.s003.docx]

**S1 Table. Assessment of measurement properties**

| Study | **Measurement tools** | **Citation for Reliability** | **Reliability Citation from Psychometric Study** | **Within Sample Reliability** | **Citation for Validity** | **Validity Citation from Psychometric Study** | **Criterion Based Validity** | **Reliability/Validity had been Established** | **Population Specific Reliability and Validity** |
| --- | --- | --- | --- | --- | --- | --- | --- | --- | --- |
| Abu-Moghli et al., 2010 | Self-report items | No | No | Yes | No | No | No | No | Reliability |
| Al-Nawaiseh et al., 2022 | Pedometer | No | No | No | No | No | No | No | No |
| BarğI, 2022 | Pedometer | Yes | Yes | No | Yes | Yes | Yes | Both | No |
|  | TheTurkey version of IPAQ-SF | Yes | Yes | No | Yes | Yes | No | Both | No |
| Barkley et al., 2017 | IPAQ | Yes | Yes | No | Yes | Yes | Yes | Both | No |
| Belogianni et al., 2023 | IPAQ-SF | No | No | No | No | No | No | No | No |
| Cameron et al., 2015 | IPAQ-SF | Yes | No | No | No | No | No | No | No |
| Choi et al., 2020 | IPAQ-SF | Yes | Yes | No | Yes | Yes | No | Both | No |
|  | Accelerometers | Yes | Yes | No | Yes | Yes | No | Both | No |
| Claxton & Wells, 2009 | The modified questionnaire from Health People 2010 | Yes | No | Yes | Yes | No | No | No | No |
| Duan et al., 2022 | The Chinese version of IPAQ-SF | Yes | Yes | No | Yes | Yes | No | No | No |
| Duan et al., 2017 | The Chinese version of IPAQ-SF | Yes | Yes | No | Yes | Yes | No | No | No |
| Eisenberg et al., 2017 | IPAQ-SF | Yes | Yes | No | Yes | Yes | Yes | Both | No |
|  | Accelerometer | Yes | Yes | No | Yes | Yes | Yes | Both | No |
| Diez et al., 2012 | HPLP-II-Spanish version | Yes | Yes | Yes | Yes | yes | No | Reliability | Yes |
| Figueroa et al., 2017 | IPAQ-SF | Yes | Yes | No | Yes | Yes | No | No | No |
| Franko et al., 2008 | **IPAQ-LF** | **Yes** | **Yes** | **Yes** | **Yes** | **Yes** | **Yes** | **Both** | **No** |
| Fukui et al., 2021 | IPAQ-SF | Yes | Yes | No | Yes | Yes | No | NO | No |
| Greene et al., 2021 | IPAQ-SF | Yes | Yes | No | Yes | Yes | No | No | No |
| Grim et al., 2021 | 7-day recall items | Yes | No | No | Yes | No | Yes | Both | No |
| Hall & Fong, 2003 | The 30-day recall measure, derived from the Stanford 7-day Recall | Yes | Yes | No | Yes | Yes | No | No | No |
| Hojjatinia et al., 2021 | Accelerometer | Yes | Yes | No | Yes | No | No | No | No |
|  | Pedometer | Yes | Yes | No | Yes | No | No | No | No |
| Kattelmann et al., 2014 | IPAQ | Yes | Yes | No | Yes | Yes | No | No | No |
| Kim et al., 2018 | Accelerometer | Yes | Yes | No | Yes | No | Yes | No | No |
| Kok et al., 2018 | IPAQ-SF | No | No | No | No | No | No | No | No |
|  | Pedometer | No | No | No | No | No | No | No | No |
| Largo-Wight et al., 2008 | Health Canada and National Quality Institute questions | No | No | No | No | No | No | No | No |
| Lee et al., 2012 | IPAQ | Yes | Yes | No | Yes | Yes | No | No | Yes |
|  | Pedometer | No | No | No | No | No | No | No | No |
| Lin et al., 2021 | The Taiwan version of IPAQ | Yes | Yes | No | Yes | Yes | No | Both | Yes |
| Loucks et al., 2021 | IPAQ | Yes | Yes | No | Yes | Yes | No | No | No |
| Lu et al., 2023 | Accelerometer | Yes | Yes | No | Yes | No | No | No | No |
| Mackey et al., 2015 | Accelerometer | Yes | Yes | No | Yes | Yes | Yes | No | No |
| Magoc et al., 2011 | IPAQ-SF | No | No | No | No | No | No | No | No |
| Marenus et al., 2021 | IPAQ-SF | Yes | Yes | Yes | Yes | Yes | Yes | Reliability | Yes |
| Martens et al., 2012 | Self-report Items | No | No | No | No | No | No | No | No |
| Maselli et al., 2019 | IPAQ-SF | Yes | Yes | No | Yes | Yes | Yes | No | No |
|  | Accelerometer | Yes | Yes | No | Yes | Yes | Yes | No | No |
| McDonough et al., 2022 | Accelerometer | No | No | No | No | No | No | No | No |
| Miragall et al., 2018 | Pedometer | Yes | Yes | No | Yes | Yes | Yes | No | No |
| Muftuler & Ince, 2015 | The Turkish version of IPAQ-SF | Yes | Yes | No | Yes | Yes | Yes | Reliability | No |
| Munoz et al., 2014 | Pedometer | No | No | No | No | No | No | No | No |
| Peng et al., 2015 | Accelerometer | Yes | Yes | No | Yes | Yes | Yes | No | No |
| Pope et al., 2019 | Accelerometer | Yes | No | No | Yes | No | No | No | No |
| Pope & Gao, 2022 | Accelerometer | Yes | No | No | Yes | No | Yes | No | No |
| Rote, 2017 | Pedometer | Yes | Yes | No | Yes | Yes | Yes | No | No |
| Ruissen et al., 2019 | GLTEQ | Yes | Yes | No | Yes | Yes | Yes | Both | No |
|  | Accelerometer | Yes | Yes | No | Yes | Yes | Yes | No | No |
| Schweitzer et al., 2016 | CCPAQ | Yes | Yes | No | Yes | Yes | No | No | No |
| Sharp & Caperchione, 2016 | The modified version of GLTEQ | Yes | Yes | Yes | Yes | Yes | Yes | No | Both |
|  | Pedometer | No | No | No | No | No | No | No | No |
| Shin et al., 2017 | The Korean Version of IPAQ | Yes | Yes | No | Yes | Yes | No | No | No |
|  | Accelerometer | No | No | No | No | No | No | No | No |
| Tulasiram & Chandrasekaran, 2021 | Pedometer | No | No | Yes | No | No | Yes | No | Both |
| Unick et al., 2017 | Pedometer | No | No | No | No | No | No | No | No |
| Yan et al., 2023 | IPAQ-SF | Yes | Yes | No | Yes | Yes | No | No | No |
| Annesi et al., 2017 | GLTEQ | Yes | Yes | No | Yes | Yes | Yes | Reliability | No |
| Brown et al., 2014 | GPAQ | Yes | Yes | No | Yes | Yes | No | No | No |
| Heeren et al., 2018 | 3 items | No | No | No | No | No | No | No | No |
| Okazaki et al., 2014 | IPAQ | Yes | Yes | No | Yes | Yes | No | No | No |
| Sriramatr et al., 2014 | The Thai Version of GLTEQ | Yes | Yes | No | Yes | Yes | No | No | No |
|  | Pedometer | No | No | No | No | No | No | No | No |

Notes: CCPAQ: Cross-Cultural Activity Patterns Questionnaire; GLTEQ: Godin Leisure-Time Exercise Questionnaire; GPAQ: Global Physical Activity Questionnaire; HPLP: Health-Promoting Lifestyle Profile; IPAQ: International Physical Activity Questionnaire; LF: long form; SF: short form.
